# Fast and Sensitive Alignment of Microbial Whole Genome Sequencing Reads to Large Sequence Datasets on a Desktop PC: Application to Metagenomic Datasets and Pathogen Identification

**DOI:** 10.1371/journal.pone.0103441

**Published:** 2014-07-31

**Authors:** Lőrinc S. Pongor, Roberto Vera, Balázs Ligeti

**Affiliations:** 1 Faculty of Information Technology, Pázmány Péter Catholic University, Budapest, Hungary; 2 2nd Department of Pediatrics, Semmelweis University, Budapest, Hungary; 3 Protein Structure and Bioinformatics Group, International Centre for Genetic Engineering and Biotechnology, Trieste, Italy; National Institutes of Health, United States of America

## Abstract

Next generation sequencing (NGS) of metagenomic samples is becoming a standard approach to detect individual species or pathogenic strains of microorganisms. Computer programs used in the NGS community have to balance between speed and sensitivity and as a result, species or strain level identification is often inaccurate and low abundance pathogens can sometimes be missed. We have developed Taxoner, an open source, taxon assignment pipeline that includes a fast aligner (e.g. Bowtie2) and a comprehensive DNA sequence database. We tested the program on simulated datasets as well as experimental data from Illumina, IonTorrent, and Roche 454 sequencing platforms. We found that Taxoner performs as well as, and often better than BLAST, but requires two orders of magnitude less running time meaning that it can be run on desktop or laptop computers. Taxoner is slower than the approaches that use small marker databases but is more sensitive due the comprehensive reference database. In addition, it can be easily tuned to specific applications using small tailored databases. When applied to metagenomic datasets, Taxoner can provide a functional summary of the genes mapped and can provide strain level identification. Taxoner is written in C for Linux operating systems. The code and documentation are available for research applications at http://code.google.com/p/taxoner.

## Introduction

Metagenomic shotgun sequencing can be used for direct taxonomic profiling of complex microbial communities, thus enabling a faster and more accurate alternative in comparison to culture-based identifications. Shotgun sequencing data analysis usually involves alignment to one large reference (e.g. the human genome), which is easily accomplished with a standard PC. Microbial shotgun sequencing data analysis is a more complex problem, since each read has to be compared to many, and sometimes millions of relatively small reference sequences which is a bottleneck for most existing analysis techniques (for reviews see [1], [2], [3]).

Current computational approaches fall into two broad categories. The first group–marker-based methods–seeks to bypass the bottleneck via search space reduction, using dedicated, small datasets. A typical example is 16S RNA analysis wherein a dataset of short sequence items is searched with sensitive alignment techniques, such as BLAST [4]. While this is the traditional standard for taxonomic identification, it has well known limitations, including the need for PCR amplification that introduces extra overhead as well as experimental bias. Alternatively, word-based techniques combined with artificial intelligence can be used to construct a database of clade-specific recognizers that make it possible to use rapid string matching techniques for species identification [5]. Finally, the MetaPhlAn program uses a small clade-specific sequence marker database built from the genome sequences of the known taxa that can be searched with general-purpose aligners. This search is extremely fast and accurate for determining taxa and their approximate proportions within large microbial communities. A potential common drawback of marker-based approaches is the frequent lack of lower (e.g. strain-level) taxon identification, as the markers are often identical for many strains. This may cause problems in identifying pathogenic strains of common commonly occurring bacteria such as *E. coli*.

In the second group of metagenome sequencing approaches, whole genome shotgun sequencing reads are directly aligned against a comprehensive sequence database. In this group of approaches database search is a critical step since aligning a large set of reads against a comprehensive database using high quality aligners such as BLAST is either too time consuming or requires computational resources that that are not readily available for all research groups. Good alternatives to BLAST style alignment are the dedicated aligners developed for next generation sequencing. Examples of these include Bowtie2 [6], BWA [7], mrFAST [8] (for a review, see [9]). These aligners are extremely fast but often require an excessive amount of memory for storing the indexed database, especially when comprehensive sequence databases are used.

A crucial step in all approaches is taxon assignment [10] which is often carried out via various flavors of lowest common ancestor search within a taxonomic hierarchy. Briefly, alignment programs assign reads either to one taxon (say, an *E. coli* strain), or to several taxa (say 100% identity with an *E. coli* strain and an *E. fergusoni* strain), and in the latter case the lowest common taxonomic ancestor (the genus Escherichia) is reported. This principle is used in popular programs such as MEGAN [5], [11], Mothur [12] and SOrt-ITEMS [13].

The variety of computational approaches available suggests that there is a need for further computational improvements. For example, the need for dedicated tools for specific challenges is clear since most of the current software tools are developed for general research purposes. A further issue is that in research settings, qualitative and quantitative answers are not always clearly separated. For instance, the presence of *E. coli* reads in an output may be a safe indication for *E. coli* being present in an environmental sample, but the number of the identified reads is not necessarily a quantitative measure of the abundance of the species. Currently, only MetaPhlAn is considered a reliable quantitative indicator for species abundance in metagenome analyses [14]. Finally, diagnostic settings pose a separate problem: here one has to precisely detect whether or not a pathogen is present above a certain threshold level, while the exact quantity is not necessarily important.

In order to address the above issues we have developed Taxoner, a simple, parallelizable pipeline that allows one to align millions of reads against the full NT database of NCBI [15] on a standard personal computer or laptop. Bowtie2 carries out the alignment and the output includes the alignments and the taxa assigned. Unlike most metagenome analysis programs, Taxoner allows identification and functional overview of the genes that received hits. We are particularly concerned with the problem of detecting low abundance species in complex datasets. This includes detecting spurious bacterial pathogens in human or animal samples. In this task, Taxoner is approximately at least as accurate as, and at times even more accurate than BLAST + MEGAN and it requires considerably (two orders of magnitude) less CPU time.

## Results

### Principle

Taxoner is a program written in C that identifies taxa, primarily bacteria, by mapping NGS reads to a comprehensive sequence database such as the NCBI NT database or its predefined subsets [15]. The program is developed so as to run on standard desktop or laptop computers under the Linux operating system.

The idea behind Taxoner comes from a technical problem. Running fast aligners such as Bowtie2 on a large number of microbial genomes is prohibitively time consuming since, at least in principle, each of the small genomes have to be indexed separately. However if we concatenate the small bacterial genomes into larger units, i.e. concatenated FASTA files that we term “artificial chromosomes”, the problem becomes more manageable. In such an artificial chromosome, a genome is a segment that is annotated by various identifiers including taxonomic name and GI identifier. As such the number of reads matching a particular genome can be counted at various taxonomic levels which corresponds to the well known principle of taxonomic binning [16]. The only prerequisite is to know the starting and endpoints of the genomes and/or other segments incorporated into the “artificial chromosome”, which is solved by pre-calculated index files.

Importantly, this process is analogous to the mapping of reads to an annotated genome wherein the segments–i.e. the genes–are named according to such schemes as COG [17], [18], GO [19] etc. Namely, in both cases, we map a read to a large sequence consisting of annotated segments, and the segments are named according to various ontologies. As a consequence, this algorithm can be used both for taxon identification and for function prediction based on NGS datasets. We highlight that mapping of counts to ontologies, sometimes also referred to as “ontology binning” is a problem known in other fields of medical informatics [20]. Further analogies can be found in protein sequence similarity searching wherein BLAST hits (HSPs) are mapped to domains annotated within proteins [21], [22], [23], [24], [25].

The algorithm consists of three phases. i) In the preprocessing phase in which a database of an arbitrary number of species is combined into partitions (“artificial chromosomes”) that are then indexed with the Bowtie2-build program of the Bowtie2 package [6]. Alternatively, pre-built indices can be downloaded from the project site (http://code.google.com/p/taxoner). ii) Alignment is then carried out with Bowtie2 and the taxa are identified with a lowest common ancestor search algorithm. The standard output of this phase is a summary of the found taxa and alignments in the SAMtools format. iii) Unlike other metagenome analysis programs Taxoner can optionally provide a list of genes identified at the species level, along with their predicted functions. It also contains a utility that can produce a summary of the found functions based on the COG-eggNog scheme of functional descriptors [17], [18] and by using a B-tree index. In addition, the read alignments provided in the SAM (Sequence Alignment/Map) format can be further processed with other taxon assignment programs such as MEGAN [11].

Details of the algorithm and datasets used are in the **Methods** section.

The source code is freely available from http://code.google.com/p/taxoner/. The program runs on Linux computers and includes a simple html graphical interface for local use. The program can be operated in the command line mode and allows evaluation of large read datasets on personal computers or laptops with at least 8 GB RAM. A demo web server, with a capacity to process datasets of up to 100,000 reads and test cases can be found at http://pongor.itk.ppke.hu/taxoner and http://pongor.itk.ppke.hu/taxoner/examples/respectively.

### Run times and examples

We evaluate the performance of Taxoner in comparison to MetaPhlAn [14], BLAST (legacy blast) [26] and BLAST + (dc-megablast) [27], both in combination with the MEGAN taxon assignment program [5], [11]. MetaPhlAn was selected because of its speed and accuracy in estimating taxon composition, while BLAST was selected because of its reputation in alignment. It is noted that comparison is a complex task since, for instance, MetaPhlAn compares reads to its own small taxon-marker database of about 367 million nucleotides that includes only bacteria. BLAST and Taxoner, on the other hand can run on comprehensive databases such as NCBI NT (52 billion nucleotides), which includes all species, or on a bacterial subset (typically 15.5 billion nucleotides). The search of the database thus impacts the speed and the accuracy of the results.

The actual alignment times for Taxoner, MetaPhlAn and BLAST depend on the size of the database and the number of threads used for the calculation, and naturally on the length and the number of the reads to be evaluated. The input read datasets used for testing are listed in **methods** and are deposited as Table S1). We carried out a number of comparisons (given as Table S1), with typical results shown in **Table 1**.

**Table 1:** Typical running times for the alignment.

		Running time^1^		
	Dbase	1 thread	4 threads	12 threads
MetaPhlAn^5^	own bacterial marker dbase^2^	14 sec	7 sec	6 sec
Taxoner^5^	NCBI nt Bacteria^3^	165 sec	105 sec	90 sec
Taxoner^5^	NCBI nt full dbase^4^	2446 sec	2031 sec	1866 sec
MEGABLAST^6^	NCBI nt bacteria^3^	8.3 h	n/a	3.9 h
MEGABLAST^6^	NCBI nt full dbase^4^	37.6 h	n/a	9.4 h

1 Read dataset: Dataset A, Table 1. Processor: Intel(R) Xeon(R) CPU E5-2640;

2 The built-in dataset is 366,988,039 nucleotides (367 MB) and contains only bacterial sequences;

3 15,400,949,699 nucleotides (15 GB), downloaded on 11/07/2013;

4 52,380,339,934 nucleotides (54 GB), downloaded on 11/07/2013;

5 Times include taxon assignment;

6 time of taxon assignment by MEGAN is not included.

The alignment times of Taxoner are thus in between those of BLAST and MetaPhlAn (see **Table 2**). Importantly, the analysis by Taxoner is fast enough to be performed on an ordinary desktop PC or on a laptop. The indexing of the database is more time consuming for Taxoner however (see **Methods**).

**Table 2:** Read assignment for *Staphylococcus aureus* genome sequencing data.

		Roche 454 (Dataset A)^1^			Ion Torrent (Dataset B)^1^			Illumina (Dataset C)^1^		
	Level:	Genus	Species	Strain	Genus	Species	Strain	Genus	Species	Strain
Taxoner	Total	93692	93692	93692	37175	37175	37175	27531	27531	27531
	Positive	93189	92728	62	36482	35919	0	26023	17019	0
	Negative	4	43	875	28	126	17174	29	121	1213
	FNR %^2^	0.004	0.046	0.934	0.075	0.339	46.198	0.105	0.440	4.406
MetaPhlAn	Total	8525	8525	8525	2522	2522	2522	1692	1692	1692
	Positive	8209	8063	N/A^3^	2402	2399	0	1650	1613	N/A
	Negative	0	42	N/A	43	36	0	2	28	N/A
	FNR %	0.000	0.493	N/A	1.705	1.427	N/A	0.118	1.655	N/A
BLASTALL + MEGAN	Total	86752	86752	86752	68696	68696	68696	45721	45721	45721
	Positive	83718	82951	156	65264	63801	0	41189	27375	0
	Negative	25	29	53	114	125	5310	408	441	3094
	FNR %	0.029	0.033	0.061	0.166	0.182	7.730	0.892	0.965	6.767
DC-MEGABLAST + Megan	Total	84211	84211	84211	64858	64858	64858	48677	48677	48677
	Positive	81000	80035	140	61662	60199	0	44161	29466	0
	Negative	48	68	131	94	128	3052	81	180	3421
	FNR %	0.057	0.081	0.156	0.145	0.197	4.706	0.166	0.370	7.028

1 100,000 random selected reads from experimental data, details in **Methods** section 4.1.

2 False Negative Rate.

3 Not available.

**Figure 1:**
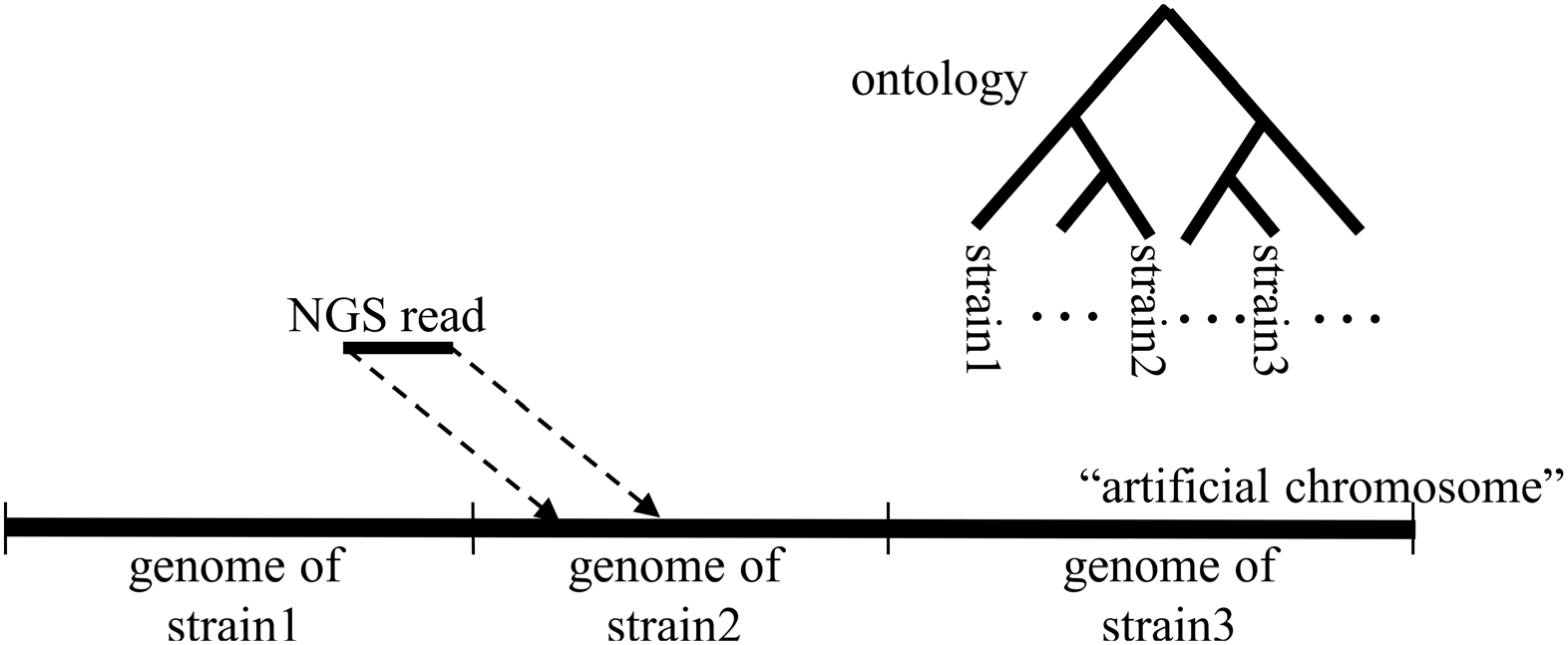
The Taxoner principle. Reads are mapped to genomes and the corresponding taxon names are read from an ontology, in this case a taxonomic tree. For function analysis, the name of the mapped gene is read from an ontology of function names such as GO.

**Figure 2:**
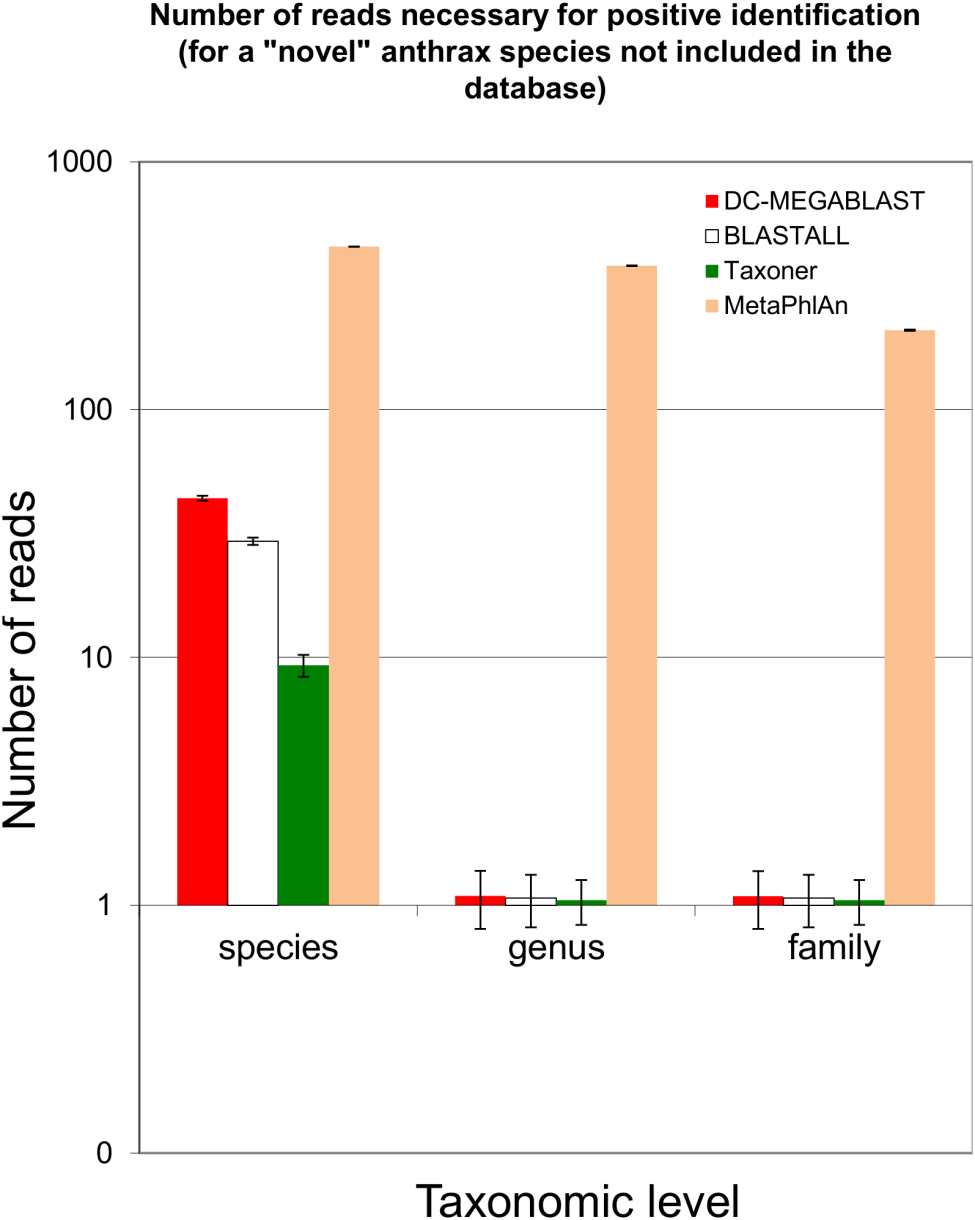
Detection of low abundance strains. Number of reads necessary on average to detect an unknown species at various taxonomy levels. Error bars indicate standard deviation of the mean, calculated from 400 repetitions.

A typical output is shown in **Figure 1**, with the reads indexed according to the NCBI taxonomy. Mapping reads to genes is provided by the Taxoner gene assignment module (see **methods**), which uses a pre-built dataset. This process typically takes more time than the alignment. Its time requirements are not included in **Table 1**. Typical results are shown in **Figure 2** (A: list of functions, B: Bar diagram).

### Compatibility with various sequencing platforms

The development of sequencing technologies has given rise to a number of sequencing platforms in recent years. The performance of read aligners often varies on the basis of reads produced by the various sequencing platforms. We compared the performance of aligner programs on read datasets selected from *Staphylococcus aureus* sequencing data (dataset A, B and C with results shown in **Table 2**). BLAST aligners had the highest alignment rate, which is not surprising since BLAST is a sensitive local aligner. MetaPhlAn had the lowest alignment rates, which is, again, expected since MetaPhlAn only aligns reads to a unique subset of the bacterial database (i.e. clade-specific markers). In general, all aligners had the best performance on the 454 dataset, since the 454 reads are longer and thus easier to analyze. All programs performed well at genus level. An important aspect of metagenome analysis is the identification of taxa at the lowest possible taxonomic levels such as species or strains. At the species level Taxoner showed the best performance with the exception of 454 reads where BLAST performed better. On the other hand, none of the programs identified strains very reliably (strain level identification is not even available with MetaPhlAn). The discrepancies may however be caused by the fact that the sequencing data used in this comparison were partly taken form uncharacterized strains (dataset B, Ion Torrent data) or from a strain not in the database of *Staphylococcus aureus* subsp. *aureus* 67–331 (NCBI taxon id: 585131, dataset C, Illumina data).

### Analyzing known and unknown strains

Given the very high sequence variability of bacterial genomes, it is crucial to know whether or not the genome of a bacterium to be detected by NGS is included in the database. This is a very important question since unknown strains represent the majority of microorganisms found in environmental samples. In the context of data analysis, a strain is known when its genome or draft genome is included in the database. With this in mind we compared the detection probability of two *Bacillus anthracis* strains (**Table 3**). The “known” strain was the Sterne strain, used for vaccination, and the dataset consisted of 100 bp long overlapping segments (“artificial reads”) of the genome, offset by 50 bp (dataset F). This is, therefore, a perfect dataset on which no mistakes are expected. The unknown strain was a Japanese isolate that was not included in the database at the time of this analysis. The dataset contained 7.7 million Illumina reads (dataset E).

**Table 3:** Analysis of known and unknown *B. anthracis* strains.

Taxa assigned		MetaPhlAn^1^	Taxoner^2^
A) Strain included in the database (*B. anthraci*s strain Sterne (NCBI taxon id: 260799), 104574 synthetic reads, Dataset F).			
All		991 reads	104,573 reads
Genus	*Bacillus*	100.00	100.00%
Species	*Bacillus anthracis*	76.60%	100.00%
Species	*Bacillus cereus*	15.40%	0.00%
Species	*Bacillus thuringiensis*	8.00%	0.00%
Species	other	0.00%	0.00%
	False negative%^3^	**23.40%**	**0.00%**
B) Strain not included in the database (*B. anthraci*s strain BA104 (NCBI taxon id: Not Available), 7.7 million Illumina reads, Dataset E)			
All		96,045 reads	7,379,118 reads
Genus	*Bacillus*	99.58%	100.00%
Species	*Bacillus anthracis*	65.30%	96.50%
Species	*Bacillus cereus*	18.70%	0.80%
Species	*Bacillus thuringiensis*	15.60%	0.40%
Species	other	0.00%	2.30%
	False negative	**34.44%**	**3.50%**

1 The values are taken from the standard output of the program.

2 Values indicate the number of reads expressed as % of the total.

3 False negative is the % of taxa (MetaPhlAn) and reads (Taxoner) detected but not present.

The number of errors detected (false negatives) was substantially higher for the strain not included in the database. The synthetic reads generated from a genome included in the database are perfectly detected by Taxoner, which is not surprising (they are perfectly detected also by BLAST, data not shown). What is somewhat unexpected is that MetaPhlAn detects a substantial % of species not present in the samples, and even in the synthetic reads. Even though we cannot explain this finding, it is good to remember that *B. anthracis* belongs to the *B. cereus* group that includes three highly related species, *B. cereus*, *B. thuringiensis* and *B. anthracis*. This is reflected by the fact that a large percentage (about 75%) of the synthetic reads generated from the Sterne strain are 100% identical in all three species (data not shown). MetaPhlAn assigns these reads to the *Bacillus* genus, while Taxoner (and MEGAN) assigns the reads to the *B. cereus* group. This illustrates the fact that species % reported by the various programs highly depend on the database as well as on the taxonomy definitions used by the given programs.

### Detection of very low abundance reads

When it comes to detecting pathogens, one of the most important questions is the sensitivity of the test, which can be estimated from the number of reads safely detected by the analysis. We tried to estimate this value by randomly selecting a fixed number of reads from an experimental anthrax dataset (dataset E) in conjunction with the NT dataset (Figure 2). MetaPhlAn was used in conjunction with its own dataset. The analysis was repeated for each set of data (see **methods** for details). It is apparent that at the species level, Taxoner/Bowtie2 performed better than BLAST or MetaPhlAn. At genus and higher levels, all methods performed well even though it was apparent that MetaPhlAn needed one-two orders of magnitude more reads to complete the identification than the other programs. Taxoner and BLAST were able to detect a genus essentially from one read while MetaPhlAn needed 200–350 reads on average for the identification. Species level identification was apparently more difficult for all programs, with Taxoner and BLAST needing 10 or 15 reads for the identification.

### Analyzing metagenomic datasets

Analysis of metagenomic data from NGS reads has two goals: a) establishing the presence or absence of taxa at the lowest possible taxonomic level and if possible at species/strain level, and b) estimating the relative amounts of taxa. We carried out an analysis of a metagenomic dataset published by the Human Microbiome Project that consisted of 6.5 and 1.4 million reads (Dataset G and H, respectively) and consisted of equal amounts of 22 strains representing 22 species. The data presented in **Table 4** show that Taxoner can detect taxa at the strain level, which is in contrast to MetaPhlAn (and other programs, such as WGSQUIKR). The accuracy of MetaPhlAn and Taxoner are comparable in this task, but it has to be mentioned that Taxoner can achieve this accuracy only if one sets a minimum threshold for the number of reads necessary to identify a taxon (strain, species, etc). MetaPhlAn uses a similar thresholding approach for improved accuracy. Without setting this threshold, Taxoner will report all spurious similarities, which would result in a very high number of false positives. In this analysis we also included WGSQUIKR, an extremely fast and innovative program that uses compressed sensing principles for finding a number of taxa that can identify the presence of the reads [28]. This analysis is extremely fast, but in our hands, the number of taxa present is apparently underestimated at all taxonomic levels.

**Table 4:** Detection of species in a metagenomic datasets.

A) Illumina sequenced HMP Mock Community sample^1^ (dataset G)													
		MetaPhlAn				Taxoner^2^				WGSQUICKR			
	No of positives (taxa present)	TP^3^	FN^4^	FP^5^	F-measure	TP	FN	FP	F-measure	TP	FN	FP	F-measure
strain	22	NA^6^	NA	NA	NA	14	7	8	0,65	1	20	79	0,02
species	22	21	1	7	0,84	20	2	0	0,95	9	13	67	0,18
genus	19	18	1	5	0,86	17	2	0	0,94	13	6	45	0,34
family	18	18	0	6	0,86	17	1	0	0,97	13	5	29	0,43
**B) 454 sequenced HMP Mock Community sample** 1 **(dataset H)**													
		**MetaPhlAn**				**Taxoner** 7				**WGSQUICKR**			
**taxa assigned**	**No of positives (taxa present)**	**TP**	**FN**	**FP**	**F-measure**	**TP**	**FN**	**FP**	**F-measure**	**TP**	**FN**	**FP**	**F-measure**
strain	22	NA	NA	NA	NA	9	12	19	0,37	1	20	58	0,03
species	22	19	3	0	0,93	19	3	0	0,93	5	17	52	0,13
genus	19	16	3	0	0,91	16	3	0	0,91	8	11	37	0,25
family	18	16	2	0	0,94	16	2	0	0,94	9	9	23	0,36

1 The data was a mock community dataset provided by the Human Microbiome Project and consisted of 22 strains.

2 Only hits (read-taxon assignments) were considered where the worst alignment score was at least 0.9. Positive taxa predicted by Taxoner are those that received at least 1000 hits (dataset G).

3 True positives.

4 False negatives.

5 False positives.

6 Not available.

7 Hits (read-taxon assignments) were only considered where the worst alignment score was at least 0.9. Positive taxa predicted by Taxoner are those that received at least 100 hits (dataset H).

### Gene and function assignment

**Figure 3:**
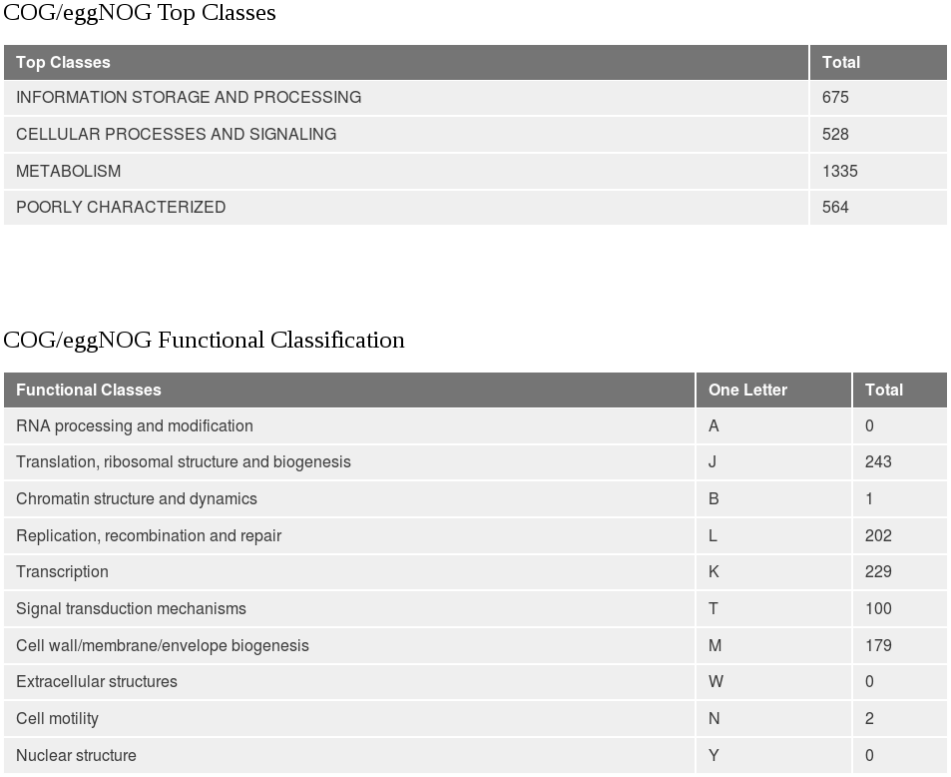
Screenshot of the Taxoner summary of gene functions. Each functional category is characterized by the number of genes that received hits.

In the Taxoner framework, detection of genes and assignment of functions is a problem analogous to function assignment (see **Figure 1**). This is a standalone utility that takes the alignment output in the Taxoner format and maps the hits to genes specified in GenBank [29]. The identifiers of the genes are stored either in a relational database, prepared with the JBioWH [30] facility, or are stored in the form of a binary file with a B-Tree index. This stored form contains the “from-to” location of each gene, the identifier and pointers to the COG-eggNog [17], [18], [31] functional classification terms. If a read is mapped to one single genome, and the hit overlaps with one or more genes, each of the concerned genes will receive one hit. If the read is 100% identical with several genomes, then the first genomes in the Bowtie2 alignment will receive the hit. After evaluating all hits in this manner, a potentially large number of genes will have hits assigned. The utility can simply list the genes with the number of hits, or can combine the genes into functional categories using the COG-eggNog scheme. As a result, the hits collected by single genes will be added up to higher categories, and graphical statistics can be made. Examples are shown in Figure 3.

### Graphical interface and demo server application

For application in pipelines, Taxoner can be run from the Unix/Linux command line. For individual users we created a graphical interface that can be installed on the user’s computer. We have provided running instructions. The same graphical interface was installed as a demo server at http://pongor.itk.ppke.hu/taxoner and this allows one to analyze default examples as well as datasets up to 50 MB of size.

## Discussion

Here we described a pipeline of programs, called Taxoner that uses a fast aligner and a comprehensive database for analyzing metagenomic datasets. As a result of alterations to the indexing used, this pipeline is fast enough to run evaluations on a single PC, and it is highly sensitive so it can be adapted to analysis of problems such as detecting pathogens in human samples. Taxoner is much faster and at times more accurate than BLAST-based evaluation schemes. In our case we tested BLAST in conjunction with the MEGAN program.

Detection of unknown strains poses problems for most aligners. It is important to remember that strains of the same species isolated from different natural environments can differ in a very large portion of their genome. As such analyzing the metagenome of soil bacteria may require the identification of strains that are largely novel as compared to the current databases. In this sense, approaches based on a comprehensive database, such as Taxoner, are at an advantage as compared to approaches based on marker databases. This is because new strains do not necessarily contain the unique sequences included in a marker database. On the other hand, this is an important problem since detection of hazardous pathogens requires strain level identification. This feature is included in Taxoner, but not in many other programs designed for metagenome analyses. We note that Taxoner uses Bowtie2 and not BLAST, resulting in its sensitivity being at times better than that of BLAST-based methodologies. This shows that fast alignment techniques combined with an appropriate database may provide a useful alternative for sensitive analysis of metagenomic samples.

Finally we note that pathogen identification is a specific task that sharply differs from metagenome analysis in many respects. Taxoner offers three possibilities that can help pathogen identification: i) The capability to filter out reads of a host organism (by specifying a host genome); ii) A Megan compatible output format that can be submitted to Megan, so that the user can manually identify expected pathogens, and iii) The possibility to use dedicated databases. For instance, the user can create a dedicated pathogen database which can then be directly used for the analysis (or simply use standard virus/pathogen databases which can be downloaded from our repository).

## Methods

The databases and datasets used in this study are listed in **Table 5**.

**Table 5:** Benchmark datasets.

Dataset	ID	Sequencing platform and number of spots; average read length	Taxon	Note
A	SRR292150	454 GS 20 (183203;110.31)	*Staphylococcus aureus* subsp.*aureus* USA300_TCH959(NCBI taxon id: 450394)	Randomly selected^1^, Supplementary file^2^
B	ERR236069	Ion Torrent PGM(1338465;262.05)	*Staphylococcus aureus*(NCBI taxon id: 1280)	Randomly selected^1^, Supplementary file^2^
C	SRR017390	Illumina Genome Analyzer II(26391487;76)	*Staphylococcus aureus* subsp. *aureus* 67–331(NCBI taxon id: 585131)	Randomly selected^1^, Supplementary file^2^
D	DRR000184	Illumina Genome Analyzer II(7631281;50)	*Bacillus anthracis* BA104 (NCBI taxon id:Not Available)	Randomly selected^1^, Supplementary file^2^
E	DRR000184	Illumina Genome Analyzer II(7631281;50)	*Bacillus anthracis* BA104 (NCBI taxon id:Not Available)	Whole run^3^
F	AE017225	NA (104574;99.99)	*Bacillus anthracis* str. Sterne(NCBI taxon id: 260799)	Full genome sampling^4^, supplementary file^2^
G	SRX055380	Illumina Genome Analyzer II(6562065; 75.00)	HMP Mock Communityeven sample	Whole genome sequencing
H	SRX030841	454 GS FLX Titanium(1386198; 530.22)	HMP Mock Communityeven sample	Whole genome sequencing

1 Random selected datasets were produced by a python script that uniformly sampled the read collection without replacement. The sample size was 100000.

2 Supplementary files are deposited at http://pongor.itk.ppke.hu/taxoner/examples/.

3 The whole run was analyzed.

4 The genome was sampled with overlapping reads. The read length was uniformly 100 bp and the overlap between the adjacent reads was 50 bp.

### The Taxoner pipeline

Taxoner is a pipeline written in C and currently uses Bowtie2 [6] (2.0.0-beta5).

The input is a reference nucleotide database in the form of concatenated FASTA file, and a set of nucleotide sequence reads typically 40–500 bp in length, in fastq format. The Taxoner database is freely available from http://pongor.itk.ppke.hu/taxoner/databases/bowtie2/. The NCBI Taxonomy retrieved on 11/07/2013 was used for taxon assignment. For function assignment, Taxoner uses a preformatted dataset that contains two binary files which can be easily created from a pipeline that uses an SQL script and the JBioWH framework [30]. The binary files can be downloaded from http://pongor.itk.ppke.hu/taxoner/databases/geneassignment/and the pipeline description can be found in the Taxoner Google Code web site.

### Algorithm outline

The algorithm performs a fast alignment with Bowtie2 against the NT (or subset of NT) database. After alignment, the lowest common ancestor for each read is determined for each database subset, which is then merged, where the final common ancestor identification is performed.

### Preprocessing

The database used for alignment is created using the NCBI NT fasta file format. The standard database creation process is done by splitting the NT fasta file into ∼4 Gb fasta files (sub-databases), where the headers of each fasta sequence are replaced with the GI identifier and the organism taxon ID. The fasta files cannot be larger than ∼4 Gb because Bowtie2 has a limit on the reference genome size. When the database is created the final step is then to index each reference fasta file with the Bowtie2-build program. Since the NT database is not restricted to microbial organisms, an alternative database can be created by extracting the subset organisms of interest (e.g. bacteria, fungi, archaea). This greatly reduces the analysis time since reads do not have to be aligned to non-microbial entries. For this reason we made a pre-parsed and indexed database for each major microbial superkingdom. This is available for download via our website at https://code.google.com/p/taxoner/wiki/07_Databases. We also included a database creator program that can create a database with any taxon ID(s) specified by the user.

### Analysis

For the analysis the user must provide the input parameters (listed and explained below, under command line usage). During analysis, Taxoner first runs Bowtie2 using the default or user specified parameters and writes the alignments into a SAM (Sequence Alignment/Map) [32] file.

The sequence alignment with Taxoner is done using the Bowtie2 aligner and the pre-indexed databases. Since the NT database is too large to fit in a single fasta file, the input sequencing reads have to be aligned separately against each sub-database. Fortunately Bowtie2 is a multithreaded aligner, which enables faster alignments using multithreaded processors. After all reads are aligned against each database, the taxonomic evaluation can be done. The main difficulty here is that a read can align with the same alignment quality to each fasta file. For this reason, Taxoner calculates a “local” common ancestor (LCA) for each read in each alignment file and then merges the results into one file. In the final step, the merged file is sorted by read name and a final LCA is calculated for each read using their “local” lowest common ancestors. The output is a file that contains read names, alignment information, nearest neighbor taxon ID, start and ending positions of the alignment against the best hit and the genome accession number of the best hit. An optional output is a MEGAN compatible output, which enables the post analysis and visualization of the results.

Function assignment is carried out by a scheme that is analogous to taxon assignment and it is performed on reads assigned to strains or to species. Briefly, a read mapping a (protein or RNA coding) gene within a strain contribute one count to that function. Reads assigned at species level are assigned to the highest-ranking gene’s function in the toplist. It is assumed that genes within a species have identical functions so if there are several hits within the same species the read is assigned to the highest-ranking annotated gene. The result of function assignment is a list of functions with the corresponding read counts. The COG-eggNog scheme of functional descriptors [17], [18] is used for functional assignments, in conjunction with a pre-calculated database file that uses the B-tree index [33] for fast function retrieval. The B-tree index algorithm was implemented in C. Its implementation is part of a C library developed by our group and is freely available at https://code.google.com/p/bioc/.

#### Output file generation

The output of this phase is a file with a list of GenBank accession number(s), taxonomy id, NCBI protein id, NCBI gene symbol, the number of reads hitting the gene, a list of COG-eggNog ids and NCBI Protein Cluster ids. Examples are deposited at http://pongor.itk.ppke.hu/taxoner/examples/.

### Command line usage

For usage in large-scale pipelines, Taxoner can be run from the command line. An example is:

./taxoner -dbPath path/to/database/fasta/−taxpath/path/to/nodes/nodes.dmp -seq/path/to/fastq/illumina.fastq -p 6 -o Results/

where the -dbPath tells Taxoner where to find the sub-databases (and Bowtie2 indexes), the -taxpath is the path to the NCBI nodes.dmp file, the -seq is the input fastq reads, -p specifies the number of threads and -o is the output folder for the results.

As an alternative, where the user wants to align reads to only a part of the database (say all the bacteria and archaea), an extra parameter −dbNames can be added, with a semicolon separated list of prefixes for each database subset:

./taxoner -dbPath path/to/database/fasta/−taxpath/path/to/nodes/nodes.dmp -seq/path/to/fastq/illumina.fastq -p 6 -o Results/−dbNames bacteria;archaea

### Graphical interface and demo server

Taxoner includes a web based graphical interface that helps users to run the programs and parse the results. This interface is a Javascript-based set of web services developed using AngularJS and running over a Nodes.JS server.

This graphical interface is designed to be used locally on a PC. It offers input forms for the different components of the Taxoner system. The starting script will open a web site running on http://127.0.0.1∶8081 (the local computer) and creates a series of web services running in the same IP address but in different ports.

It is well know that modern web browsers do not have access to the local file system due to a security issue. As such a set of different web services must be created to allow access to the local files from the web browser.

The Taxoner web services are running on ports from 8081 to the 8084. The port 8081 is for the HTTP web site (the user interface). The rest are ports for internal uses: port 8082 offer a web service to read and parse the log files of Taxoner, 8083 is a web service to run system commands from the web browser and 8084 is a web service to create the Taxoner summaries from the output files.

Three web forms are available on the graphical interface–a form to run the Taxoner program, a form to run the gene assignment program and a form to parse the outputs of both programs and show a summary of them.

Full description and images of the graphical interface can be seen at https://code.google.com/p/taxoner/wiki/06_Graphical.

In addition, a demo server was developed for demonstration purposes using the same look and feel of the local graphical interface. This web server offers the same features as the graphical interface but has a file size limitation for the input files. The demo server is available from http://pongor.itk.ppke.hu/taxoner.

### Parameter settings and other programs used for comparison

The performance and accuracy of Taxoner was compared with several popular mapping tools using the following parameter settings:

- MetaPhlAn [14] (with the recommended parameter settings)
- legacy BLAST (blastall program with the default parameter settings)
- BLAST + (blastn program with dc-megablast task with the default parameter settings)
- WGSQUIKR [28] (run with the default parameter settings)
- Taxoner (this work) (default parameters)

## Supporting Information

Table S1 List of files used for comparison.
(DOCX)

## References

[1] Teeling H, Glockner FO (2012) Current opportunities and challenges in microbial metagenome analysis–a bioinformatic perspective. Brief Bioinform 13: 728–742. 22966151 10.1093/bib/bbs039 PMC3504927

[2] Kunin V, Copeland A, Lapidus A, Mavromatis K, Hugenholtz P (2008) A bioinformatician’s guide to metagenomics. Microbiol Mol Biol Rev 72: 557–578. 19052320 10.1128/MMBR.00009-08 PMC2593568

[3] Neelakanta G, Sultana H (2013) The Use of Metagenomic Approaches to Analyze Changes in Microbial Communities. Microbiol Insights 6: 37–48. 24826073 10.4137/MBI.S10819 PMC3987754

[4] Altschul SF, Madden TL, Schaffer AA, Zhang J, Zhang Z, et al (1997) Gapped BLAST and PSI-BLAST: a new generation of protein database search programs. Nucleic Acids Res 25: 3389–3402. 9254694 10.1093/nar/25.17.3389 PMC146917

[5] Huson DH, Richter DC, Mitra S, Auch AF, Schuster SC (2009) Methods for comparative metagenomics. BMC Bioinformatics 10 Suppl 1: S12. 10.1186/1471-2105-10-S1-S12 PMC2648729 19208111

[6] Langmead B, Salzberg SL (2012) Fast gapped-read alignment with Bowtie 2. Nat Methods 9: 357–359. 22388286 10.1038/nmeth.1923 PMC3322381

[7] Li H, Durbin R (2010) Fast and accurate long-read alignment with Burrows-Wheeler transform. Bioinformatics 26: 589–595. 20080505 10.1093/bioinformatics/btp698 PMC2828108

[8] Hach F, Hormozdiari F, Alkan C, Birol I, Eichler EE, et al (2010) mrsFAST: a cache-oblivious algorithm for short-read mapping. Nat Methods 7: 576–577. 20676076 10.1038/nmeth0810-576 PMC3115707

[9] Li H, Homer N (2010) A survey of sequence alignment algorithms for next-generation sequencing. Brief Bioinform 11: 473–483. 20460430 10.1093/bib/bbq015 PMC2943993

[10] Droge J, McHardy AC (2012) Taxonomic binning of metagenome samples generated by next-generation sequencing technologies. Brief Bioinform 13: 646–655. 22851513 10.1093/bib/bbs031

[11] Huson DH, Auch AF, Qi J, Schuster SC (2007) MEGAN analysis of metagenomic data. Genome Res 17: 377–386. 17255551 10.1101/gr.5969107 PMC1800929

[12] Schloss PD, Westcott SL, Ryabin T, Hall JR, Hartmann M, et al (2009) Introducing mothur: open-source, platform-independent, community-supported software for describing and comparing microbial communities. Appl Environ Microbiol 75: 7537–7541. 19801464 10.1128/AEM.01541-09 PMC2786419

[13] Monzoorul Haque M, Ghosh TS, Komanduri D, Mande SS (2009) SOrt-ITEMS: Sequence orthology based approach for improved taxonomic estimation of metagenomic sequences. Bioinformatics 25: 1722–1730. 19439565 10.1093/bioinformatics/btp317

[14] Segata N, Waldron L, Ballarini A, Narasimhan V, Jousson O, et al (2012) Metagenomic microbial community profiling using unique clade-specific marker genes. Nat Methods 9: 811–814. 22688413 10.1038/nmeth.2066 PMC3443552

[15] NCBI Resource Coordinators (2014) Database resources of the National Center for Biotechnology Information. Nucleic Acids Res 42: D7–17. 24259429 10.1093/nar/gkt1146 PMC3965057

[16] Dröge J, McHardy AC (2012) Taxonomic binning of metagenome samples generated by next-generation sequencing technologies. Brief Bioinform 13: 646–655. 22851513 10.1093/bib/bbs031

[17] Powell S, Forslund K, Szklarczyk D, Trachana K, Roth A, et al (2014) eggNOG v4.0: nested orthology inference across 3686 organisms. Nucleic Acids Res 42: D231–239. 24297252 10.1093/nar/gkt1253 PMC3964997

[18] Tatusov RL, Fedorova ND, Jackson JD, Jacobs AR, Kiryutin B, et al (2003) The COG database: an updated version includes eukaryotes. BMC Bioinformatics 4: 41. 12969510 10.1186/1471-2105-4-41 PMC222959

[19] Ashburner M, Ball CA, Blake JA, Botstein D, Butler H, et al (2000) Gene Ontology: tool for the unification of biology. Nat Genet 25: 25–29. 10802651 10.1038/75556 PMC3037419

[20] Moore CB, Wallace JR, Frase AT, Pendergrass SA, Ritchie MD (2013) BioBin: a bioinformatics tool for automating the binning of rare variants using publicly available biological knowledge. BMC Med Gen 6: S6. 10.1186/1755-8794-6-S2-S6 PMC3654874 23819467

[21] Dhir S, Pacurar M, Franklin D, Gáspári Z, Kertész-Farkas A, et al (2010) Detecting atypical examples of known domain types by sequence similarity searching: The SBASE domain library approach. Curr Protein Pept Sci 11: 538–549. 20887262 10.2174/138920310794109148

[22] Murvai J, Vlahovicek K, Barta E, Parthasarathy S, Hegyi H, et al (1999) The domain-server: direct prediction of protein domain-homologies from BLAST search. Bioinformatics 15: 343–344. 10320404 10.1093/bioinformatics/15.4.343

[23] Lu S, Deng P, Liu X, Luo J, Han R, et al (1999) Solution structure of the major alpha-amylase inhibitor of the crop plant amaranth. J Biol Chem 274: 20473–20478. 10400675 10.1074/jbc.274.29.20473

[24] Maravic G, Bujnicki JM, Feder M, Pongor S, Flogel M (2003) Alanine-scanning mutagenesis of the predicted rRNA-binding domain of ErmC’ redefines the substrate-binding site and suggests a model for protein-RNA interactions. Nucleic Acids Res 31: 4941–4949. 12907737 10.1093/nar/gkg666 PMC169915

[25] Maravic G, Feder M, Pongor S, Flogel M, Bujnicki JM (2003) Mutational analysis defines the roles of conserved amino acid residues in the predicted catalytic pocket of the rRNA: m6A methyltransferase ErmC’. J Mol Biol 332: 99–109. 12946350 10.1016/s0022-2836(03)00863-5

[26] Shiryev SA, Papadopoulos JS, Schaffer AA, Agarwala R (2007) Improved BLAST searches using longer words for protein seeding. Bioinformatics 23: 2949–2951. 17921491 10.1093/bioinformatics/btm479

[27] Zhang Z, Schwartz S, Wagner L, Miller W (2000) A greedy algorithm for aligning DNA sequences. J Comput Biol 7: 203–214. 10890397 10.1089/10665270050081478

[28] Koslicki D, Foucart S, Rosen G (2014) WGSQuikr: Fast Whole-Genome Shotgun Metagenomic Classification. PloS one 9: e91784. 24626336 10.1371/journal.pone.0091784 PMC3953531

[29] Benson DA, Clark K, Karsch-Mizrachi I, Lipman DJ, Ostell J, et al (2014) GenBank. Nucleic Acids Res 42: D32–37. 24217914 10.1093/nar/gkt1030 PMC3965104

[30] Vera R, Perez-Riverol Y, Perez S, Ligeti B, Kertesz-Farkas A, et al (2013) JBioWH: an open-source Java framework for bioinformatics data integration. Database (Oxford) 2013: bat051. 23846595 10.1093/database/bat051 PMC3708619

[31] Jensen LJ, Julien P, Kuhn M, von Mering C, Muller J, et al (2008) eggNOG: automated construction and annotation of orthologous groups of genes. Nucleic Acids Res 36: D250–254. 17942413 10.1093/nar/gkm796 PMC2238944

[32] Li H, Handsaker B, Wysoker A, Fennell T, Ruan J, et al (2009) The sequence alignment/map format and SAMtools. Bioinformatics 25: 2078–2079. 19505943 10.1093/bioinformatics/btp352 PMC2723002

[33] Bayer R, McCreight E (1972) Organization and Maintenance of Large Ordered Indexes. Acta Informatica 1: 173–189.

