# Supplementary material for: Fast and Sensitive Alignment of Microbial Whole Genome Sequencing Reads to Large Sequence Datasets on a Desktop PC: Application to Metagenomic Datasets and Pathogen Identification
Source: PLoS One. 2014 Jul 31;9(7):e103441. doi: 10.1371/journal.pone.0103441 (PMC4117525; doi:10.1371/journal.pone.0103441)
Supplement: Table S1 — List of files used for comparison. (DOCX) [file pone.0103441.s001.docx]

**Supplementary File1**

| SRA ID | Sequencing platform and  number of spots; average read length | Taxon | Note | File name |
| --- | --- | --- | --- | --- |
| SRR292150 | 454 GS 20 (183203;110.31) | Staphylococcus aureus subsp. aureus USA300_TCH959 (NCBI taxon id: 450394) | Randomly selected | 454_100000sample.zip |
| ERR236069 | Ion Torrent PGM (1338465;262.05) | Staphylococcus aureus (NCBI taxon id: 1280) | Randomly selected | iontorrent_100000sample.zip |
| SRR017390 | Illumina Genome Analyzer II (26391487;76) | Staphylococcus aureus subsp. aureus 67-331 (NCBI taxon id: 585131) | Randomly selected | illumine_100000sample.zip |
| DRR000184 | Illumina Genome Analyzer II (7631281;50) | Bacillus anthracis BA104 (NCBI taxon id: Not Available) | Randomly selected | anthrax_100000sample.zip |

Table S1. List of files used for comparison
